# Floating Thrombus on the Ascending Aorta and/or Aortic Arch, to Operate or Not to Operate: Two Case Reports and a Literature Review

**DOI:** 10.3390/jcdd12070248

**Published:** 2025-06-27

**Authors:** Estelle Demoulin, Jalal Jolou, Raoul Schorer, Bernhard Walder, Carl Glessgen, Christoph Huber, Mustafa Cikirikcioglu

**Affiliations:** 1 Division of Cardiovascular Surgery, Department of Surgery, University Hospital Geneva, Rue Gabrielle-Perret-Gentil 4, CH-1205 Genève, Switzerland; estelle.demoulin@hug.ch (E.D.);; 2 Division of Peri-Interventional Care, Department of Anaesthesiology-Intensive Care and Pharmacology, University Hospital Geneva, Rue Gabrielle-Perret-Gentil 4, CH-1205 Genève, Switzerland; 3 Division of Radiology, Department of Diagnostic, University Hospital Geneva, Rue Gabrielle-Perret-Gentil 4, CH-1205 Genève, Switzerland

**Keywords:** floating aortic thrombus, aortic thrombus, systemic embolism, surgical thrombectomy, endovascular management, aortic embolism

## Abstract

Background and Aim: Floating aortic thrombi are rare but potentially life-threatening entities, associated with a high risk of systemic embolization and subsequent complications such as ischemic stroke or mesenteric infarction. Therapeutic strategies range from urgent surgical intervention to conservative medical management with anticoagulation, depending on the patient’s clinical status and thrombus morphology. This report presents two cases of floating aortic thrombi managed with distinct approaches, surgical and medical, underscoring the importance of individualized treatment guided by embolic risk and comorbidities. Patients and Methods: The first case involves a 59-year-old male presenting with abdominal pain and emesis. Imaging confirmed mesenteric ischemia, necessitating emergent laparotomy and extensive jejunal resection. Postoperative imaging identified a mobile thrombus at the ascending aorta–aortic arch junction, with evidence of cerebral embolism. The patient underwent urgent surgical thrombectomy, ascending aortic resection, and hemiarch replacement. The second case describes an 88-year-old male who presented with bilateral upper limb paresthesia. Neuroimaging revealed acute supra- and infratentorial ischemic lesions suggestive of embolic stroke. A floating thrombus was identified in the ascending aorta, with an additional thrombus in the descending thoracic aorta. Given the patient’s advanced age, comorbid conditions, and thrombus stability, a conservative approach with systemic anticoagulation and close radiologic surveillance was chosen. Conclusions: These cases illustrate the need for tailored management of floating aortic thrombi. While surgical resection remains indicated in unstable or high-risk embolic cases, anticoagulation may suffice for stable lesions in patients with elevated surgical risk. Further studies are needed to establish standardized therapeutic guidelines.

## 1. Introduction

Floating aortic thrombi (FAT) represent a rare but clinically significant pathology [1], particularly when located in the ascending aorta or aortic arch. These intraluminal thrombi carry a substantial risk of systemic embolization, potentially resulting in catastrophic complications such as ischemic stroke, mesenteric infarction, peripheral limb ischemia, or other visceral organ damage [2]. Although uncommon, the optimal management of FAT remains a subject of debate, with therapeutic strategies ranging from emergent surgical thrombectomy to conservative medical therapy with systemic anticoagulation. The choice of intervention is guided by multiple factors, including thrombus morphology, location, mobility, evidence of embolization, and the patient’s overall physiological status and comorbid conditions.

The pathogenesis of FAT is believed to involve the classical components of Virchow’s triad (endothelial injury, hypercoagulability, and aberrant hemodynamics). Despite the high-velocity, laminar flow characteristic of the aorta, thrombus formation can occur when localized disruptions, such as atherosclerotic plaques, ulcerated lesions, or aneurysmal segments, create favorable conditions for thrombus adherence. Additionally, systemic prothrombotic states associated with malignancies, autoimmune diseases, or chronic inflammatory conditions may further predispose to intraluminal thrombogenesis.

Due to the rarity of FAT and the absence of large-scale, randomized controlled trials, there is no universally accepted treatment algorithm. While some advocate for prompt surgical intervention to mitigate the risk of embolic complications, others support a conservative approach with anticoagulation in cases where the thrombus is stable and surgical risk is prohibitive. This therapeutic divergence underscores the importance of individualized patient assessment and multidisciplinary decision-making.

In this report, we describe two illustrative cases of FAT. The first involves a 59-year-old patient who underwent urgent surgical resection of the thrombus following embolic complications, including mesenteric ischemia and cerebral infarction. The second case concerns an 88-year-old patient managed conservatively with anticoagulation, considering thrombus stability and the absence of acute ischemic events.

## 2. Patient and Methods

This review presents two clinical cases of floating aortic thrombus (FAT) managed at our institution, combined with a review of the current literature to contextualize diagnostic and therapeutic strategies.

Case Data Collection: Clinical, imaging, laboratory, surgical, and follow-up data for both patients were collected retrospectively from the electronic medical records of our hospital. All patient data were anonymized, and consent was obtained.

Literature Search Strategy: To support our discussion, a literature search was conducted using PubMed databases, covering publications up to May 2025. The following search terms were used in combination:

- MeSH Terms: Aorta, Thrombosis, Embolism, Aortic Disease;
  Filters were applied to select articles in English and human studies only.

Inclusion Criteria:

- Case reports, case series, retrospective or prospective studies;
- Articles describing diagnosis, management, and outcomes of FAT;
- Studies including either surgical or medical management.

Exclusion Criteria:

- Isolated aortic mural thrombi without evidence of mobility;
- Incomplete case descriptions or animal studies;
- Abstract-only publications without full text available.

The most relevant studies were included to illustrate current trends, therapeutic options, and controversies in FAT management.

## 3. Patient 1: Surgical Management of Floating Aortic Thrombus

A 59-year-old male with a medical history notable for active tobacco use, excessive alcohol intake, hypercholesterolemia, and arterial hypertension presented to the emergency department with a two-week history of abdominal pain accompanied by episodes of vomiting.

At day 0 of presentation of symptoms, contrast-enhanced abdominal imaging revealed findings consistent with mesenteric ischemia and extensive intestinal necrosis. The patient was taken emergently on the same day to the operating room, where an exploratory laparotomy was performed, resulting in resection of approximately two meters of non-viable jejunum.

**Figure 1:**
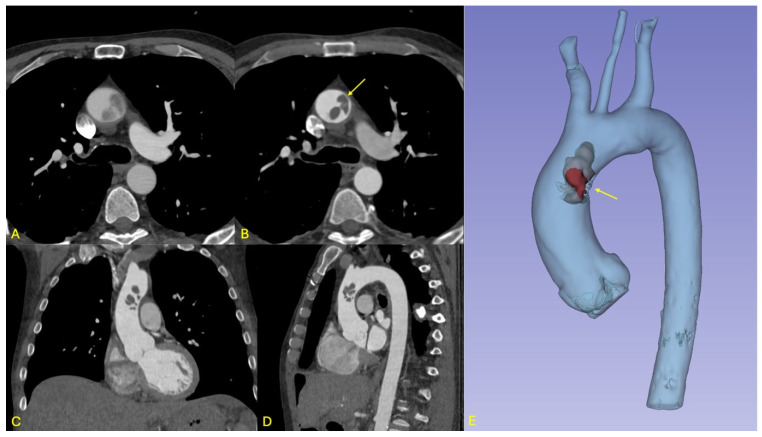
Overview of imaging findings in patient 1. (**A**,**B**): Respective axial views from the non-gated and ECG-gated CT angiographies performed at initial imaging work-up showing the impact of ECG-gating on thrombus visualization and contours. (**A**,**B**) Lobulated endoluminal structure with no apparent contact with the aorta is visualized on this slice (arrow in **B**). (**C**,**D**): Coronal and sagittal views of the ECG-gated CT angiography showing the cranio-caudal extension of this thrombotic formation. (**E**): 3D multi-planar reconstruction showing the significance of the thrombus with respect to the aortic lumen, and the attachment to the left aspect of the ascending aorta (arrow). No other thrombi were identified.

In the immediate postoperative period, a few hours after the surgery, the patient developed new-onset neurological deficits, including expressive aphasia and right upper limb paresis. A non-contrast cranial computed tomography (CT) scan demonstrated a subacute ischemic infarct in the left middle cerebral artery (MCA) territory. On day 1 after the surgery, subsequent contrast-enhanced whole-body CT imaging revealed a mobile thrombus located at the junction of the ascending aorta and the aortic arch, representing a critical embolic source with potential for both cerebrovascular and visceral embolization (see Figure 1).

Given the high embolic potential of the aortic thrombus, a multidisciplinary team discussion took place within 24 h of diagnosis and concluded that urgent surgical intervention was warranted. The patient was brought to the operating room and placed under general anesthesia in supine position. Following standard aseptic preparation and draping, a formal surgical time-out was conducted, and antibiotic prophylaxis was administered per institutional protocol.

A median sternotomy was performed, followed by pericardiotomy and meticulous dissection of the ascending aorta, brachiocephalic trunk, right subclavian artery, right common carotid artery, and left common carotid artery. Systemic anticoagulation was initiated with intravenous heparin. Tangential clamping of the right subclavian artery was carried out, allowing the anastomosis of an 8 mm Dacron graft to this vessel. An arterial cannula was then inserted into the graft, and a dual-stage venous cannula was placed into the right atrium. Cardiopulmonary bypass (CPB) was initiated, and systemic cooling was commenced to a target temperature of 32 °C. A left atrial suction cannula was introduced via the right superior pulmonary vein.

To facilitate selective cerebral perfusion, the left carotid artery was ligated, and the brachiocephalic trunk was clamped. The ascending aorta was opened without cross-clamping to minimize the risk of thrombus dislodgement and distal embolization. Cardioplegia (Cardioplexol) was administered selectively into the left and right coronary arteries, achieving diastolic cardiac arrest.

**Figure 2:**
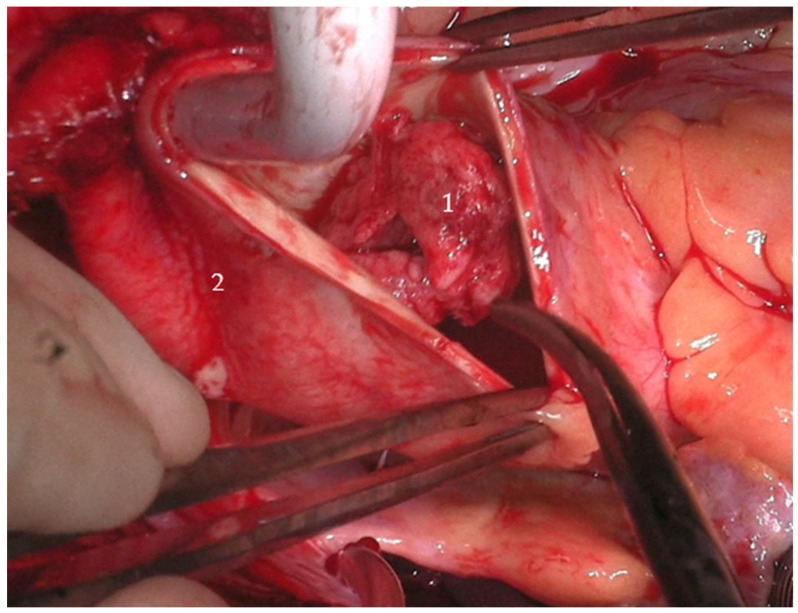
Thrombus visualization during surgery of patient 1. 1: floating aortic thrombus. 2: ascending aorta.

Two large, mobile thrombi (each approximately 3 cm × 4 cm) were identified in the distal ascending aorta, associated with ulcerated aortic wall segments. Additional ulcerations were noted at the proximal aortic arch (see Figure 2). The affected aortic segments were excised, and a 28 mm Dacron graft was used to perform a hemiarch replacement using an open distal anastomosis technique. After securing the distal anastomosis, vascular clamps were applied to the graft, cerebral perfusion was re-established, and systemic circulation was resumed. The patient was then gradually rewarmed to 36 °C.

The proximal anastomosis between the Dacron graft and the aortic root was constructed using a continuous 5-0 Prolene suture. Following careful de-airing of the graft, the aortic clamp was released, and the heart resumed spontaneous sinus rhythm at normothermia. The patient was successfully weaned from CPB with stable hemodynamics. Intraoperative transesophageal echocardiography confirmed satisfactory graft function and no residual thrombus.

Heparin reversal was achieved with protamine, and meticulous hemostasis was performed. Two temporary epicardial pacing wires were placed, and three drains were inserted into the retrosternal, retrocardiac, and right pleural spaces. The pericardium was closed using 2-0 Vicryl sutures, and the sternum was closed with steel wires. Layered closure of the soft tissues was performed, and sterile dressings were applied.

The patient’s postoperative course was uneventful, marked by rapid extubation and progressive clinical improvement in the intensive care unit. Given his stable hemodynamic status, he was transferred to the neurology ward for continued care and monitoring. Further investigations were initiated to determine the etiology of the floating aortic thrombus, raising a strong suspicion of hepatocellular carcinoma (HCC) as a potential underlying cause. As the patient was a visiting Irish national temporarily in Switzerland, he preferred to return to Ireland to pursue further diagnostic workup and oncologic evaluation. Once medically stable, he was repatriated without complication.

This case underscores the diagnostic challenge of identifying floating aortic thrombi (FAT) in the setting of nonspecific gastrointestinal and neurological presentations. In this instance, the diagnosis was made only after the occurrence of severe ischemic complications, including mesenteric infarction and ischemic stroke. These findings highlight the potential value of early vascular imaging in patients presenting with unexplained or recurrent embolic phenomena. The temporal proximity between abdominal and cerebral ischemic events suggests a high embolic load and emphasizes the urgency of prompt intervention.

Intraoperatively, the surgical team faced significant technical complexity due to the size, mobility, and friability of the thrombus. The decision to avoid cross-clamping of the aorta prior to opening was a key maneuver to minimize the risk of iatrogenic distal embolization. This approach facilitated neuroprotection while ensuring complete thrombus excision, demonstrating the critical importance of preoperative planning and multidisciplinary coordination in managing FAT.

The suspicion of hepatocellular carcinoma postoperatively raises the plausible hypothesis of a malignancy-associated hypercoagulable state contributing to thrombus formation [3]. Although definitive oncologic diagnosis was not possible following the patient’s transfer, the clinical context supports the consideration of a paraneoplastic etiology. Neoplastic processes are well-documented risk factors for thromboembolic events, largely due to tumor-driven activation of coagulation pathways. Malignant cells can secrete procoagulant factors such as tissue factor and cancer procoagulant, directly stimulating thrombin generation. Additionally, the release of inflammatory cytokines [4] can induce endothelial dysfunction, further promoting thrombogenesis.

This case illustrates the relevance of Trousseau’s syndrome, also known as malignancy-associated migratory thrombophlebitis, a paraneoplastic phenomenon frequently linked with pancreatic, gastric, and pulmonary carcinomas. It exemplifies how underlying malignancies can generate a systemic hypercoagulable state, leading to diverse thromboembolic events, including intravascular thrombi in high-flow vessels such as the aorta.

In summary, this case highlights the importance of early recognition and timely surgical intervention in patients with floating aortic thrombi, particularly in the context of ongoing or recurrent embolic events. It also reinforces the necessity of investigating occult malignancy as a potential etiological factor in unexplained thrombotic presentations, especially in patients without a history of cardiovascular disease.

## 4. Patient 2: Conservative Management of Floating Aortic Thrombus

An 88-year-old male presented to the emergency department with bilateral hand paresthesia, raising clinical suspicion for an acute cerebrovascular event. His medical history was notable for a prior right temporal ischemic stroke in 2021. Neurological examination revealed a right homonymous hemianopia and mild hemiparesis, corresponding to a National Institutes of Health Stroke Scale (NIHSS) score of 2.

**Figure 3:**
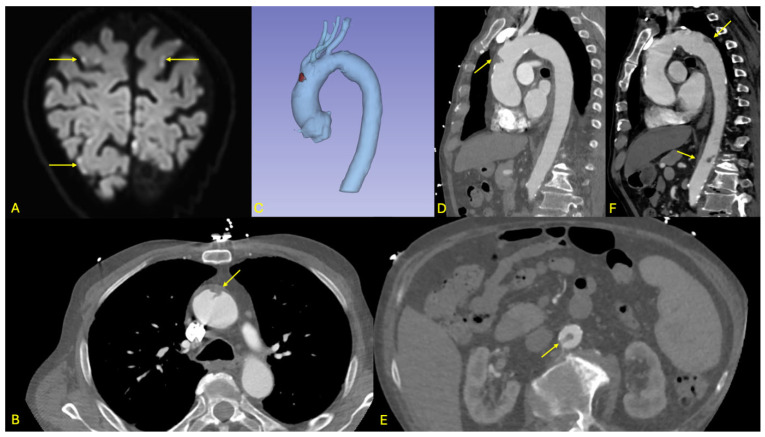
Overview of initial and follow-up imaging findings in patient 2. (**A**): Diffusion-weighted MRI sequence displayed in oblique coronal view showing multiple dot-like cortical hyperintensities of the posterior and pre-central regions (arrows), consistent with embolic stroke. (**B**–**D**): Axial and sagittal views as well as 3D multiplanar reconstruction from the initial ECG-gated CT angiography of the aorta, showing an endoluminal thrombus attached to a soft-plaque (arrow) of the anterior aspect of the ascending aorta and bulging into the aortic lumen. (**E**): Axial view from the initial aortic CT angiography showing a second, more tubular thrombus of the subrenal aorta (arrow). (**F**): Follow-up CTA showing regression of the thrombus in the ascending aorta, as well as two new thrombi (arrows), in front of the isthmus and at the thoraco-abdominal junction; the subrenal thrombus had resolved.

Brain magnetic resonance imaging (MRI) demonstrated multiple acute ischemic lesions involving both supratentorial and infratentorial regions, with some foci exhibiting hemorrhagic transformation. The distribution of lesions across multiple vascular territories was suggestive of an embolic etiology, although the exact timing of the ischemic events remained unclear (see Figure 3).

Initial non-contrast CT imaging incidentally revealed a floating thrombus within the aortic arch, prompting further evaluation with an urgent contrast-enhanced CT angiography. The angio-CT confirmed the presence of a stable, mobile thrombus located in the distal ascending aorta (see Figure 3). Additionally, the scan identified a small ulcerated atherosclerotic plaque in the abdominal aorta just proximal to the origin of the celiac trunk, as well as a second floating thrombus in the subrenal segment of the abdominal aorta. No evidence of ischemia was detected in the solid intra-abdominal organs, and the splanchnic arterial branches remained patent.

Following multidisciplinary team (MDT) consultation, surgical intervention was deferred considering the thrombi’s radiological stability, the absence of critical ischemic symptoms, and the patient’s advanced age and frailty. Anticoagulation therapy was initiated with low-molecular-weight heparin (LMWH) and subsequently transitioned to long-term oral anticoagulation using vitamin K antagonists (VKAs). International normalized ratio (INR) levels were closely monitored to maintain therapeutic anticoagulation and mitigate hemorrhagic risk.

The patient underwent serial clinical evaluations and imaging follow-up. A repeat CT angiography at three months demonstrated no significant morphological changes in either thrombus, nor was there any evidence of new embolic events or ischemic sequelae. Based on these findings, the MDT reaffirmed the conservative management strategy, with continuation of anticoagulation and without surgical intervention.

This case exemplifies the complex therapeutic balancing required in elderly patients presenting with embolic stroke and concurrent hemorrhagic transformation. The initial use of LMWH, followed by a cautious transition to VKAs, reflected an effort to control thrombus progression while minimizing bleeding risk. The presence of multiple, spatially distinct aortic thrombi raises the possibility of a systemic prothrombotic state, although no underlying thrombophilia or malignancy was identified during the index hospitalization. A full thrombophilia panel was not pursued due to the patient’s age and clinical stability.

The favorable clinical course, characterized by thrombus stability and absence of embolic recurrence, supports the feasibility of conservative medical management in select high-risk patients. However, this outcome underscores the importance of meticulous INR monitoring, regular imaging, and coordinated multidisciplinary oversight. Ultimately, this case highlights the value of an individualized, risk-adapted approach in managing floating aortic thrombi, particularly in elderly, comorbid patients without overt ischemic manifestations.

## 5. Discussion

To conclude, floating aortic thrombus (FAT) is a rare clinical entity, with an estimated incidence of approximately 0.45% in the general population. It is defined by the presence of a thrombus that is not fully adherent to the aortic wall, allowing free movement within the aortic lumen. The descending aorta is the most frequently affected site, followed by the aortic arch, while involvement of the ascending aorta is less common [5], likely due to the high velocity and laminar nature of blood flow in this region.

The etiology of FAT is multifactorial. Common predisposing factors include atherosclerosis, hypercoagulable states, malignancies, as illustrated in our first patient, chronic inflammatory diseases, trauma, and previous surgical procedures. Nevertheless, FAT may also occur in patients without identifiable risk factors, rendering its pathophysiology in such cases poorly understood [6].

Given its propensity to cause severe systemic embolization, including ischemic stroke, visceral infarction, and peripheral arterial occlusion, early recognition and prompt management are essential. Therapeutic strategies are individualized and typically depend on thrombus morphology, location, embolic risk, and patient-specific characteristics, ranging from systemic anticoagulation to emergent surgical resection.

In this review, we reported two contrasting cases. The first involved a 59-year-old male who initially presented with abdominal pain and vomiting, and subsequently developed neurological deficits following emergency bowel resection for mesenteric ischemia. Imaging revealed a left Sylvian ischemic stroke and a large floating thrombus in the ascending aorta. The second case concerned an 88-year-old male with a history of ischemic stroke, who presented with bilateral hand paresthesias. Imaging revealed both supra- and infratentorial ischemic lesions and stable floating thrombi in the ascending and subrenal abdominal aorta, without evidence of acute visceral injury.

A review of the literature supports this dichotomy. Multiple authors, including Yang et al. [7] and Choi et al. [8], emphasize the importance of surgical intervention in cases involving large, mobile thrombi or when ischemic complications have already occurred. Yang et al. [6] described surgical excision of a large aortic arch thrombus with high embolic risk, analogous to our first case. Similarly, Choi et al. [8] reported successful surgical removal of a thrombus extending into the left subclavian artery, with favorable outcomes. Both cases underscore the role of early surgery in preventing recurrent embolic events.

Noh et al. [9] and Weiss et al. [10] also documented cases where surgery was preferred due to high thrombus mobility and associated embolic risk. Noh et al. [9] reported a mobile thrombus in the aortic arch causing peripheral embolization, necessitating surgical excision. Weiss et al. [10], in a series of 10 patients, showed that surgical thrombectomy was both effective and safe, with no thrombus recurrence or embolic events during follow-up. These studies reinforce the rationale for our surgical approach in the first case.

Conversely, several authors support conservative management in selected cases. Christou et al. [11] reported a patient with a large, mobile thrombus in the aortic arch managed successfully with anticoagulation due to elevated surgical risk, mirroring our management of the second patient. Parato et al. [12] likewise documented favorable long-term outcomes with anticoagulation in high-risk or elderly patients. These findings support the safety and efficacy of non-surgical treatment in well-selected, stable individuals.

Furthermore, Li et al. [13] and Majdi et al. [14] highlighted the necessity of individualized treatment plans. Li et al. [13] described surgical resection of a thrombus spanning the ascending aorta and arch due to high embolic potential, while Majdi et al. [14] reported two cases of successful surgical intervention for symptomatic FAT. These studies affirm that while surgery remains the gold standard in large or symptomatic cases, anticoagulation may be adequate in less severe presentations.

Risk stratification remains a challenge in FAT management. Some authors have proposed using thrombus mobility, anatomical location, size, and clinical context to guide therapeutic decisions; however, no standardized criteria or scoring system currently exist. Notably, thrombi in the ascending aorta or arch are generally considered to pose higher embolic risk than those in the descending aorta, often justifying more aggressive treatment. Similarly, the presence of embolic complications typically shifts the balance toward surgical intervention.

Long-term outcomes remain difficult to predict. For conservatively managed patients, recurrence rates and optimal anticoagulation duration are not well established. Some studies recommend indefinite anticoagulation, particularly in patients with persistent risk factors. Furthermore, quality-of-life outcomes and functional recovery following treatment are underreported, highlighting a gap in patient-centered research.

An additional area of uncertainty pertains to the natural history of FAT. Because many cases are diagnosed incidentally or after embolic complications, the interval between thrombus formation and clinical manifestation is poorly understood. This limits the development of effective early detection strategies. Prospective registries and longitudinal studies may help clarify the incidence, progression, and risk factors associated with FAT.

On the other hand, endovascular approaches have recently emerged as a minimally invasive alternative, particularly for thrombi located in the descending aorta. Fueglistaler et al. [15] reported successful exclusion of a symptomatic mobile thrombus in the thoracic aorta using an endovascular stent graft. Nguyen et al. [16] also reviewed therapeutic strategies for descending aortic thrombi and concluded that covered stent grafts are a feasible and safe alternative in patients not suitable for conservative therapy. Rivera et al. [17] demonstrated a high success rate and safety profile of endovascular techniques in both thoracic and abdominal aortic thrombi, supporting their consideration as first-line treatment in select cases.

Transesophageal echocardiography (TEE) also plays a critical role in the diagnostic and therapeutic landscape of aortic pathologies. While often performed for cardiac indications, TEE may lead to the incidental detection of aortic thrombi or complex atheromatous plaques, especially in patients evaluated for embolic strokes or endocarditis. Moreover, TEE provides high-resolution imaging of the thoracic aorta and is invaluable for intraoperative monitoring during both open surgical and endovascular procedures involving the aorta. Its real-time capabilities allow for the assessment of thrombus mobility, device placement, and early detection of procedural complications. As highlighted in the recent review by Trimarchi and Al [18], the integration of TEE into both diagnostic workflows and interventional planning can improve outcomes and reduce embolic risk in patients with FAT.

Overall, the literature confirms that the management of FAT must be tailored to the individual patient.

Another important consideration in the management of floating aortic thrombi is the establishment of an appropriate follow-up strategy. Given the risk of recurrence, progression, or delayed embolization, close clinical and radiological surveillance is essential, particularly in patients managed conservatively. Current practice typically involves serial contrast-enhanced CT angiography at 1, 3, and 6 months post-diagnosis, followed by annual imaging if stability is confirmed. In patients on anticoagulation, regular INR monitoring (for those on VKAs) or assessment of anti-Xa levels (for those on LMWH) is necessary to ensure therapeutic efficacy and minimize bleeding risk.

In addition, ongoing evaluation for potential underlying etiologies, such as occult malignancy, thrombophilia, or systemic inflammatory conditions, may be warranted in patients without clear risk factors. Functional follow-up, including neurological and vascular assessments, is also recommended for patients who have experienced embolic complications.

Given the absence of standardized guidelines, a multidisciplinary approach, involving cardiology, cardiac/vascular surgery, hematology, and radiology, is crucial to tailoring follow-up protocols based on individual risk profiles. Future studies should aim to define evidence-based surveillance intervals and identify prognostic indicators that can guide the intensity and duration of follow-up in this heterogeneous patient population.

## 6. Conclusions

Floating aortic thrombi are uncommon but carry a substantial risk of systemic embolization, warranting prompt recognition and a patient-specific management approach. The therapeutic decision between surgical resection and anticoagulation must be guided by thrombus morphology, anatomical location, patient comorbidities, and overall embolic risk. Our review highlights the critical importance of individualized care pathways in optimizing clinical outcomes.

Nonetheless, the current lack of standardized management guidelines, validated risk stratification tools, and robust long-term outcome data presents a major challenge. Multicenter prospective studies and international registries are urgently needed to establish evidence-based recommendations. Future research should also focus on the development of advanced imaging modalities, biomarkers predictive of thrombus instability, and clinical risk assessment models to support therapeutic decision-making.

In the interim, multidisciplinary team discussions and shared decision-making remain the foundational principles for the safe and effective management of FAT, ensuring that treatment strategies are both tailored to each patient’s situation.

## Data Availability

Details were harvested from our local software with patient information.
